# ﻿Diet composition and interspecific niche of Taohongling Sika deer (*Cervusnipponkopschi*) and its sympatric Reeve’s muntjac (*Muntiacusreevesi*) and Chinese hare (*Lepussinensis*) in winter (Animalia, Mammalia)

**DOI:** 10.3897/zookeys.1149.96936

**Published:** 2023-02-22

**Authors:** Dandan Wang, Xiaolong Hu, Minling Li, Jie Liu, Ming Tang, Wuhua Liu, Jianwen Zhan, Yongtao Xu, Weiwei Zhang

**Affiliations:** 1 College of Forestry, Wildlife Conservation Research Center, Jiangxi Agricultural University, Nanchang, Jiangxi, China; 2 College of Animal Science and Technology, Jiangxi Agricultural University, Nanchang, Jiangxi, China; 3 Taohongling Sika Deer National Nature Reserve, Pengze, Jiangxi, China

**Keywords:** Diet composition, niche breadth, niche overlap, sympatry, winter

## Abstract

Species co-existence depends on how organisms utilize their environment and resources. Little is known about the winter diet composition and sympatric co-existence of South China sika deer and its companion species in Taohongling. In this study, high-throughput sequencing and metabarcoding trnL were used to study the diet composition and interspecific relationship including sika deer, Reeve’s muntjac, and Chinese hare. Our results show that 203 genera in 90 families are contained in the diet of sika deer, 203 genera in 95 families for Reeve’s muntjac, and 163 genera in 75 families for Chinese hare. Sika deer fed on *Rubuschingii*, *Loropetalumchinense*, and *Euryajaponica* in winter, accounting for 75.30%; Reeve’s muntjac consumed mainly *R.chingii*, *E.japonica*, and *Euonymusgrandiflorus*, accounting for 68.80%, and Chinese hare mainly fed on *R.chingii*, *Smilaxchina*, and *Rhuschinensis*, accounting for 41.98%. The Shannon index showed no significant difference between groups (*p* > 0.05). The NMDS analysis found considerable overlap among three species. Sika deer and Reeve’s muntjac consumed similar forage plants but varied greatly in Chinese hare, which occupied the widest choice in winter, resulting in higher diet breadth and increased dietary divergence, thereby reducing competition and facilitating coexistence. The diet niche overlap index among them, as represented by Pianka’s index, ranging from 0.62 between sika deer and Chinese hare to 0.83 between sika deer and Reeve’s muntjac, which indicated a more similar niche and potential competition in closely related species. Our findings provide a new diet perspective of three herbivores, leading to a more comprehensive understanding of resource partitioning and species coexistence.

## ﻿Introduction

Sika deer (*Cervusnippon* Temminck, 1838), also known as the spotted deer, is a species native to much of East Asia and a national first-class protected wild animal in China (Yao et al. 2010). Its conservation status is Endangered globally and Endangered in China (Wang and Song 2013). Most wild sika deer populations in China have disappeared due to heavy hunting pressure that has existed for a very long period (Zhang et al. 2011a), and populations have also become gradually more isolated. South China sika deer (*Cervusnipponkopschi* Swinhoe, 1873) are mainly distributed in the Taohongling Sika deer National Nature Reserve (hereafter, TNNR) in northeastern Jiangxi Province, southern Anhui Province, and a part of northwestern Zhejiang Province. Statistics have previously shown that the population of sika deer in TNNR is 365 individuals (Gao et al. 2009; Jiang et al. 2012) which inhabit the hilly area at 300–500 m elevation (Wang 2018). Reeve’s muntjac and Chinese hare are the main companion species of sika deer, and they have co-existed in the TNNR for numerous generations. Infrared-camera detection has been used to determine that the relative abundance of the Reeve’s muntjac was 0.4160, which is significantly higher than that of the sika deer population (0.0411), and the relative abundance of the Chinese hare was 0.0138. Furthermore, the spatial distribution of both sika deer and Reeve’s muntjac is primarily concentrated in the core area (Zhou 2019). Nowadays, little is known about the diets and sympatric co-existence of these three herbivores, particularly in winter when their food resources are scarcer.

Diet analysis is one of the core contents of studying the habitat requirements of animals (Liu 2009; Hoenig et al. 2022). Food not only provides the necessary energy and nutrients for life activities but also reflects the trophic niche of the species in the biome (Lu et al. 2020). Therefore, diet analysis can serve to understand a species’ access to resources and habitat distribution to facilitate population conservation and recovery of endangered species. The study of diet mainly includes stomach contents analysis, indirect utilization methods, direct tracking observation, microscopic fecal analysis, and DNA metabarcoding analysis (Monro 1982; Zheng and Bao 2004; Lu et al. 2020). Stomach contents analysis is more accurate for identifying food resources but collecting stomach contents requires sacrificing animals (Fujii et al. 2019). Utilization methods and tracking observations are difficult to observe and may be influenced by subjective factors (Gong et al. 2022). Fecal microscopic analysis can be quantitative (Zhang et al. 2011b) but requires accurate identification of taxa from partially digested plant fragments and likely over-emphasizes less digestible components of the diet (Holechek 1982). For the endangered species, it is necessary to prioritize noninvasive sampling. Diet research must adopt proven selection methods based on actual needs and conditions.

High-throughput sequencing (HTS) has the advantages of high throughput, a large amount of data, high sensitivity, and fine classification (Pompanon et al. 2012; Deagle et al. 2019). Compared with conventional methods of diet research, the metabarcoding method based on high-throughput sequencing can improve the deficiencies of the traditional methods that do not fully reflect consumers’ diet information (Ma et al. 2021; Tabassum et al. 2022). The present technique has achieved remarkable results in diet research on filter-feeding shellfish (Kasim and Mukai 2009), small herbivores, fish, etc. (Soininen et al. 2009; Lin et al. 2018; Li et al. 2021), and this can identify species of lower taxonomic orders.

Food resources are the medium that connects the natural environment and often influence the distribution and survival of species. Species coexistence theory suggests that niche overlap and potential competition will inevitably occur when closely related species with similar ecological needs share the same area, which requires them to obtain more resources to survive by expanding the niche scale (Schaller 2000; Palmer and Truscott 2003). We predict that the three species in this study have been sympatric and have evolved together for numerous generations in the TNNR. Natural selection may have led to a separation in forage use (niche differentiation) among them (Pascual-Rico et al. 2020). Fitness may be reduced by competition; i.e., sika deer may increase their niche breadth, particularly in winter when food resources are scarcer (Schoener 1971). We explore the diet composition and dietary overlap of Taohongling Sika deer, Reeve’s muntjac, and Chinese hare and assess the extent of potential dietary competition among these species to enhance our understanding of mechanisms underlying their coexistence. Research into the diet of sika deer and its sympatric herbivores can clarify food items and explore the interspecific competition and coexistence, which is of great significance to the population conservation of sika deer and biodiversity monitoring.

## ﻿Methods

### ﻿Study area

**Figure 1.**
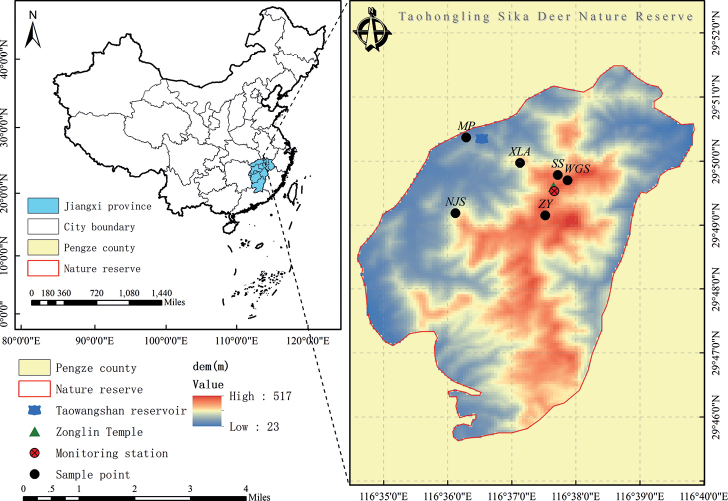
Fecal samples sites of three sympatric species at Taohongling Sika Deer Nature Reserve (MP: Nursery bases; SS: Fir forests; NJS: NieJiashan; XLA: XianLingAn; WGS: WuGuiShi; ZY: Bamboo Garden).

This study was conducted in the TNNR, the area where South China sika deer is distributed. The TNNR is located on the south bank of the middle and lower reaches of the Yangtze River, Pengze, Jiangxi Province, China. The total area of TNNR is 12,500 hm^2^, the core area is 2,670 hm^2^, the experimental area is 1,830 hm^2^, and the buffer zone is 8,000 hm^2^. The TNNR mainly consists of low mountains and hills (Wang et al. 2021). The TNNR lies in a climatic zone transitional from tropical to middle subtropical and has transitional climate characteristics that are warm, with a humid monsoon, and four distinct seasons. The frost-free period is up to 247 days with little snow cover (Zhou 2019). The vegetation type is mainly composed of mixed evergreen–deciduous broad-leaved forest, coniferous forest, mixed coniferous–broad-leaved forest, broad-leaved forest, and bamboo (Zhou 2019). From December 2020 to February 2021, 90 fecal samples were collected from sika deer, Reeve’s muntjac, and Chinese hare in the TNNR and stored at −80 °C. Sampling sites mainly focused on Nursery bases (MP), Fir forests (SS), NieJiashan (NJS), XianLingAn (XLA), WuGuiShi (WGS), and Bamboo Garden (ZY) (Fig. 1).

### ﻿DNA extraction and PCR amplification

In our study, to minimize possible bias caused by variation in individual digestibility, five fecal pellets were randomly taken from each fecal sample and mixed to form a single composite sample. Total DNA was extracted using the DNA extraction kit (TIANGEN, Beijing) following the liquid nitrogen grinding method. The final DNA concentration and purification were determined by NanoDrop 2000 UV-vis spectrophotometer (Thermo Scientific, Wilmington, USA), and DNA quality was checked by 1% agarose gel electrophoresis. The P6 loop region of the trnL(UAA)intron region was amplified with universal primers g (5՚-GGGCAATC CTGAGCCAA-3՚) and h (5՚-CCATTGAGTCTCTGCACCTATC-3՚) by thermocycler PCR system (Gene Amp 9700, ABI, USA). PCR amplifications were carried out in a total volume of 25 μl containing 12.5 μl PCR mix (Tiangen, Beijing, China), 1 μl DNA, 1 μl of each primer, and 9.5 μl H_2_O. The reaction conditions were as follows: denaturation at 95 °C for 3 min followed by 35 cycles at 95 °C for 30 sec, 56 °C for 30 sec, and 72 °C for 45 sec, with a final 10 min at 72 °C and storage at 4 °C for 10 h. The PCR products were detected by Agarose gel electrophoresis and sequenced by Shanghai Personal Biotechnology Co., Ltd.

### ﻿Illumina MiSeq sequencing and bioinformatics analysis

Purified amplicons were pooled in equimolar and paired end sequenced (2 × 300) on an Illumina MiSeq platform (Illumina, San Diego, USA) according to the standard protocols. The analysis was conducted by following the tutorial of QIIME2 docs along with customized program scripts (https://docs.qiime2.org/2019.1/). Briefly, raw FASTQ files were demultiplexed using the QIIME2 v. 2019.4 demux plugin based on their unique barcodes (Caporaso et al. 2010). Demultiplexed sequences from each sample were quality filtered and trimmed, denoised, and merged, and then the chimeric sequences were identified and removed using the QIIME2 dada2 plugin to obtain the feature table of operational taxonomic units (OTUs). The QIIME2 feature-classifier plugin was then used to align OTUs sequences to the National Center for Biotechnology Information (NCBI) database to generate the taxonomy table. Diversity metrics were calculated using the core-diversity plugin within QIIME2. Feature level alpha diversity indices, such as observed OTUs, Chao1 richness estimator, and Goods coverage index were calculated to estimate the diet diversity within an individual sample. A difference significance test was performed by R v. 4.1.3. Beta diversity distance measurements using Bray–Curtis were performed to investigate the structural variation of fecal plant communities across samples and then visualized via principal coordinate analysis (PCoA) and nonmetric multidimensional scaling (NMDS) (Vázquez-Baeza et al. 2013). Venn diagram analysis was performed to explore the common and special OTUs types among the three herbivores.

### ﻿Data statistics

Formulas of forage plants diversity and niche analysis were conducted as follows:

The Shannon–Wiener diversity index (*H*′) was calculated to explore diet diversity (Shannon 1948), according to the following formula:

$H^{'}=-\sum_{i=1}^{n}P_{i}\ln P_{i}$ (1)

Where *P_i_* is the proportion of food item *i* out of all foods, and *n* is the total number of food items.

Pielou evenness index (Pielou 1969), according to the following formula:

*J*′ = *H′ / H*_max_ (2)

*H*_max_ = ln *n* (3)

Where *n* is the number of plant species in the fecal sample, and the number of plant species is represented by the number of plant OTUs types.

The Levin index (Smith 1982) was applied to standardize the trophic niche measure, and the formula was as follows:

$B=1/\sum_{i=1}^{S}p_{i}^{2}$ (4)

The niche overlap index was obtained using the Pianka index (Pianka 1973; Hou et al. 2021), and the formula was as follows:

$Q_{jk}=\frac{\sum_{i=1}^{S}P_{ij}P_{ik}}{\sqrt{\sum_{i=1}^{S}P_{ij}^{2}\sum_{i=1}^{S}P_{ik}^{2}}}$ (5)

Where *Q_jk_* is Pianka’s niche overlap index between species *j* and species *k*; *P_ij_* is the proportion of resource *i* out of all resources used by species *j*, and *P_ik_* is the proportion of resource *i* out of all resources used by species *k*. The values range from 0 (no food item in common) to 1 (complete overlap in resource use).

## ﻿Results

### OTUs analysis

**Figure 2.**
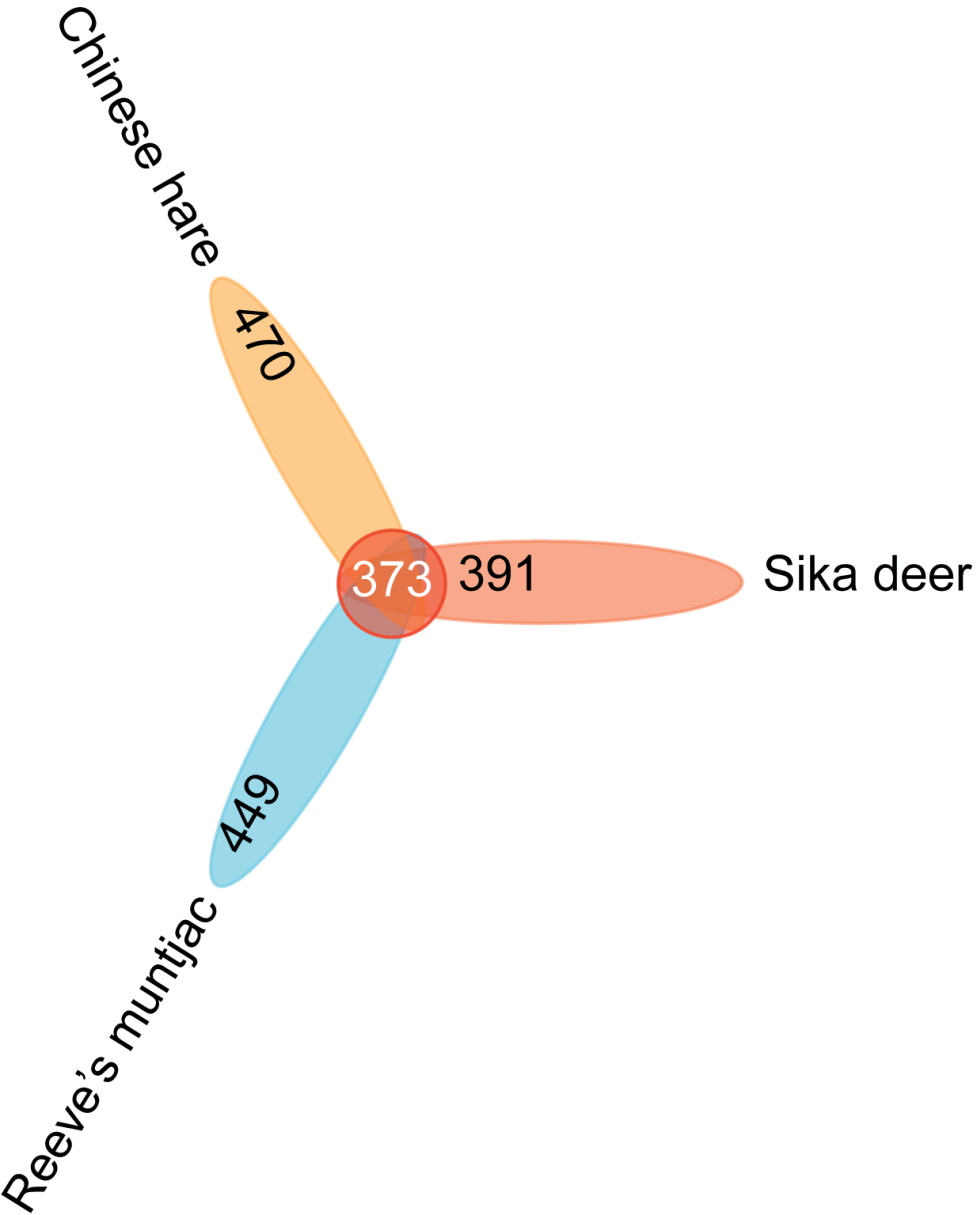
Venn analysis of OTUs in three herbivores of Taohongling nature reserve.

After processing the raw reads, a total of 11,411,958 counts were obtained from 90 fecal samples. The mean OTUs length was 67.96 bp with a range from 32 bp to 189 bp. Venn diagram showed that the OTUs in the overlap were commonly shared, and those in the nonoverlapping parts were special OTUs. In total, 764 OTUs, 833 OTUs, and 843 OTUs were obtained from sika deer, Reeve’s muntjac, and Chinese hare samples, respectively. The number of OTUs among the three herbivore groups was 373, and the specific OTUs in sika deer, Reeve’s muntjac, and Chinese hare were 391, 449, and 470, respectively (Fig. 2).

Based on OTUs sequences alignment in the NCBI database, the diets of sika deer, Reeve’s muntjac, and Chinese hare includes 203 genera in 90 families, 203 genera in 95 families, and 163 genera in 75 families, respectively (Editorial Committee of FRPS 2004; Jiang 2009; see Suppl. material 1). The three species consumed common and specific forage plants but varied greatly in their use of the available forages. On the whole, the 10 most abundant families in the diet of sika deer include Rosaceae (36.94%), Hamamelidaceae (25.75%), Pentaphylacaceae (13.41%), Theaceae (3.89%), Celastraceae (3.01%), Poaceae (2.98%), Ericaceae (2.43%), Moraceae (2.09%), Cupressaceae (1.68%), and Cannabaceae (1.16%). For the Reeve’s muntjac, the 10 most abundant families include Rosaceae (52.41%), Pentagliaceae (11.01%), Celastraceae (6.12%), Poaceae (4.52%), Cannabaceae (3.27%), Moraceae (2.64%), Sabiaceae (2.14%), Asteraceae (2.10%), Oleaceae (1.50%), and Smilacaceae (1.26%), and the 10 most abundant families in the Chinese hare consist of Rosaceae (16.35%), Poaceae (16.33%), Smilacaceae (15.58%), Anacardiaceae (10.71%), Fabaceae (9.74%), Asteraceae (6.77%), Rubiaceae (5.56%), and Cupressaceae (4.49%).

**Figure 3.**
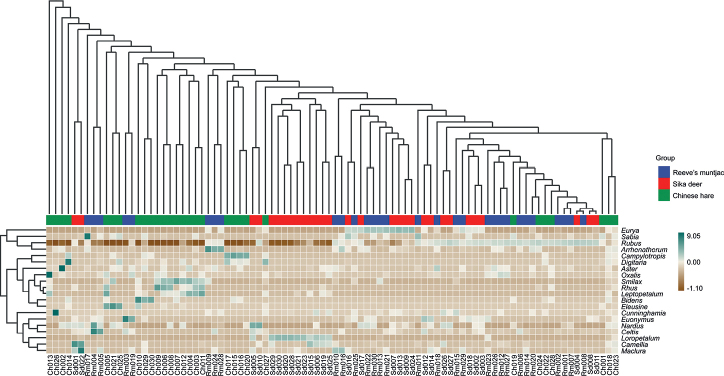
Species composition heat map at the genus level. (Ch: Chinese hare; Sd: Sika deer; Rm: Reeve’s muntjac).

The dominant genera foraged by sika deer were *Rubus* (36.49%) and *Loropetalum* (25.52%), followed by *Eurya* (13.41%), *Camellia* (3.89%), *Euonymus* (2.94%), *Phyllostachys* (2.46%), *Maclura* (1.88%), *Cunninghamia* (1.67%), *Rhododendron* (1.45%), and *Celtis* (0.99%), and others (9.30%). For Reeve’s muntjac the diet was strongly dominated by *Rubus* (51.77%), other genera high abundance were *Eurya* (11.01%), *Euonymus* (6.11%), *Celtis* (3.21%), *Arrhenatherum* (3.02%), *Sabia* (2.14%), *Maclura* (1.67%), *Ligustrum* (1.49%), *Phyllostachys* (1.31%), and *Smilax* (1.26%). Chinese hare consumed almost equal proportions of *Rubus* (15.81%) and *Smilax* (15.58%), followed by *Rhus* (10.64%), *Campylotropis* (9.52%), *Bidens* (5.37%), *Hedyotis* (5.02%), *Cunninghamia* (4.47%), *Eleusine* (3.57%), *Digitaria* (3.09%) and *Miscanthus* (2.55%). To sum up, these three herbivores all mostly feed on *Rubus* in winter. The species composition heatmap was drawn from the species and sample levels. The 20 genera with the highest abundance were selected based on species annotation information for 90 samples of the three species. The clustering results show the differences in the relative abundances of sika deer, Chinese hare, and Reeve’s muntjac (Fig. 3).

High-throughput sequencing can be used to detect the diet on species levels in most samples combined with a background survey of TNNR, except for OTUs that were undetectable. Sika deer food items included *Rubuschingii*, *Loropetalumchinense*, *Euryajaponica*, *Camelliajaponica*, *Euonymusgrandiflorus*, etc. The forage plants of Reeve’s muntjac consisted of *Rubuschingii*, *Euryajaponica*, *Euonymusgrandiflorus*, *Arrhenatherumelatius*, *Celtissinensis*, etc. The diet of Chinese hares mainly focused on *Rubuschingii*, *Smilaxchina*, *Rhuschinensis*, *Campylotropis* sp., *Hedyotisdiffusa*, etc. The detailed top 30 forage species in three species are shown in Table 1.

**Table 1.** Winter diet of sika deer, Reeve’s muntjac, and Chinese hare in Taohongling nature reserve. “+” = present in Jiangxi; “–” = not or uncertain present in Jiangxi.

Number	Sika deer				Reeve’s muntjac				Chinese hare			
	Species	Genus	Disrtibution	Percentage of abundance	Species	Genus	Distribution	Percentage of abundance	Species	Genus	Distribution	Percentage of abundance
1	*Rubuschingii*	*Rubus*	+	36.42%	*Rubuschingii*	*Rubus*	+	51.69%	*Rubuschingii*	*Rubus*	+	15.78%
2	*Loropetalumchinense*	*Loropetalum*	+	25.48%	*Euryajaponica*	*Eurya*	+	11.01%	*Smilaxchina*	*Smilax*	+	15.56%
3	*Euryajaponica*	*Eurya*	+	13.41%	*Euonymusgrandiflorus*	*Euonymus*	+	6.10%	*Rhuschinensis*	*Rhus*	+	10.64%
4	*Camelliajaponica*	*Camellia*	+	3.88%	*Arrhenatherumelatius*	*Arrhenatherum*	+	3.02%	*Campylotropis* sp.	*Campylotropis*	–	9.40%
5	*Euonymusgrandiflorus*	*Euonymus*	+	2.94%	*Celtissinensis*	*Celtis*	+	2.57%	*Bidens* sp.	*Bidens*	–	5.37%
6	*Phyllostachysedulis*	*Phyllostachys*	+	2.46%	*Sabiaswinhoei*	*Sabia*	+	2.14%	*Hedyotisdiffusa*	*Hedyotis*	+	5.02%
7	*Macluratricuspidata*	*Maclura*	+	1.88%	*Macluratricuspidata*	*Maclura*	+	1.66%	*Cunninghamialanceolata*	*Cunninghamia*	+	4.47%
8	*Cunninghamialanceolata*	*Cunninghamia*	+	1.67%	*Ligustrumlucidum*	*Ligustrum*	+	1.48%	*Eleusineindica*	*Eleusine*	+	3.57%
9	*Rhododendronmucronatum*	*Rhododendron*	+	1.45%	*Phyllostachysedulis*	*Phyllostachys*	+	1.31%	*Digitaria* sp.	*Digitaria*	–	2.79%
10	*Vacciniumvitis-idaea*	*Vaccinium*	+	0.77%	*Smilaxchina*	*Smilax*	+	1.26%	*Miscanthussinensis*	*Miscanthus*	+	2.55%
11	*Celtissinensis*	*Celtis*	+	0.78%	*Cunninghamialanceolata*	*Cunninghamia*	+	1.21%	*Oxaliscorniculata*	*Oxalis*	+	2.31%
12	*Ilexcornuta*	*Ilex*	+	0.59%	*Nyssa* sp.	*Nyssa*	–	1.16%	*Setariaviridis*	*Setaria*	+	1.56%
13	*Sabiaswinhoei*	*Sabia*	+	0.48%	*Aster* sp.	*Aster*	–	1.08%	*Panicumbisulcatum*	*Panicum*	+	1.49%
14	*Cocculusorbiculatus*	*Cocculus*	+	0.37%	*Loropetalumchinense*	*Loropetalum*	+	0.83%	*Pinusmassoniana*	*Pinus*	+	1.46%
15	*Lysimachiacongestiflora*	*Lysimachia*	+	0.32%	*Lysimachiacongestiflora*	*Lysimachia*	+	0.81%	*Aster* sp.	*Aster*	–	1.18%
16	*Saxifragastolonifera*	*Saxifraga*	+	0.30%	*Oxaliscorniculata*	*Oxalis*	+	0.79%	*Secalecereale*	*Secale*	+	1.16%
17	*Arrhenatherumelatius*	*Arrhenatherum*	+	0.27%	*Ilexcornuta*	*Ilex*	+	0.67%	*Polypogonfugax*	*Polypogon*	+	1.00%
18	*Ehretiaacuminata*	*Ehretia*	+	0.27%	*Celtis* sp.	*Celtis*	–	0.64%	*Phyllostachysedulis*	*Phyllostachys*	+	0.96%
19	*Nyssa* sp.	*Nyssa*	–	0.22%	*Cirsiumarvense*	*Cirsium*	+	0.62%	*Sabiaswinhoei*	*Sabia*	+	0.96%
20	*Hedyotisdiffusa*	*Hedyotis*	+	0.21%	*Broussonetiapapyrifera*	*Broussonetia*	+	0.59%	*Euryajaponica*	*Eurya*	+	0.73%
21	*Rhuschinensis*	*Rhus*	+	0.22%	*Saxifragastolonifera*	*Saxifraga*	+	0.44%	*Mallotusjaponicus*	*Mallotus*	+	0.59%
22	*Celtis* sp.	*Celtis*	–	0.20%	*Rhododendronmucronatum*	*Rhododendron*	+	0.40%	*Liquidambarformosana*	*Liquidambar*	+	0.55%
23	*Corylopsismultiflora*	*Corylopsis*	+	0.20%	*Mallotusjaponicus*	*Mallotus*	+	0.37%	*Nicotianatabacum*	*Nicotiana*	+	0.55%
24	*Polypogonfugax*	*Polypogon*	+	0.19%	*Tetradiumruticarpum*	*Tetradium*	+	0.32%	*Loropetalumchinense*	*Loropetalum*	+	0.49%
25	*Abeliaschumannii*	*Abelia*	+	0.18%	*Galiumaparine*	*Galium*	+	0.32%	*Prunussibirica*	*Prunus*	–	0.46%
26	*Broussonetiapapyrifera*	*Broussonetia*	+	0.15%	*Coreopsistinctoria*	*Coreopsis*	+	0.29%	*Arrhenatherumelatius*	*Arrhenatherum*	+	0.39%
27	*Vacciniumovalifolium*	*Vaccinium*	–	0.15%	*Leptodermis* sp.	*Leptodermis*	–	0.25%	*Ilexcornuta*	*Ilex*	+	0.38%
28	*Pinusmassoniana*	*Pinus*	+	0.14%	*Iryantherahostmannii*	*Iryanthera*	–	0.25%	*Sargentodoxacuneata*	*Sargentodoxa*	+	0.32%
29	*Pteroceltistatarinowii*	*Pteroceltis*	+	0.14%	*Morusyunnanensis*	*Morus*	+	0.24%	*Rhododendronmucronatum*	*Rhododendron*	+	0.29%
30	*Oxaliscorniculata*	*Oxalis*	+	0.12%	*Acorusgramineus*	*Acorus*	+	0.24%	*Microstegiumvimineum*	*Microstegium*	+	0.28%
31	Others			4.14%	Others			6.24%	Others			7.74%

### ﻿Diet diversity and interspecific niche analysis

**Figure 4.**
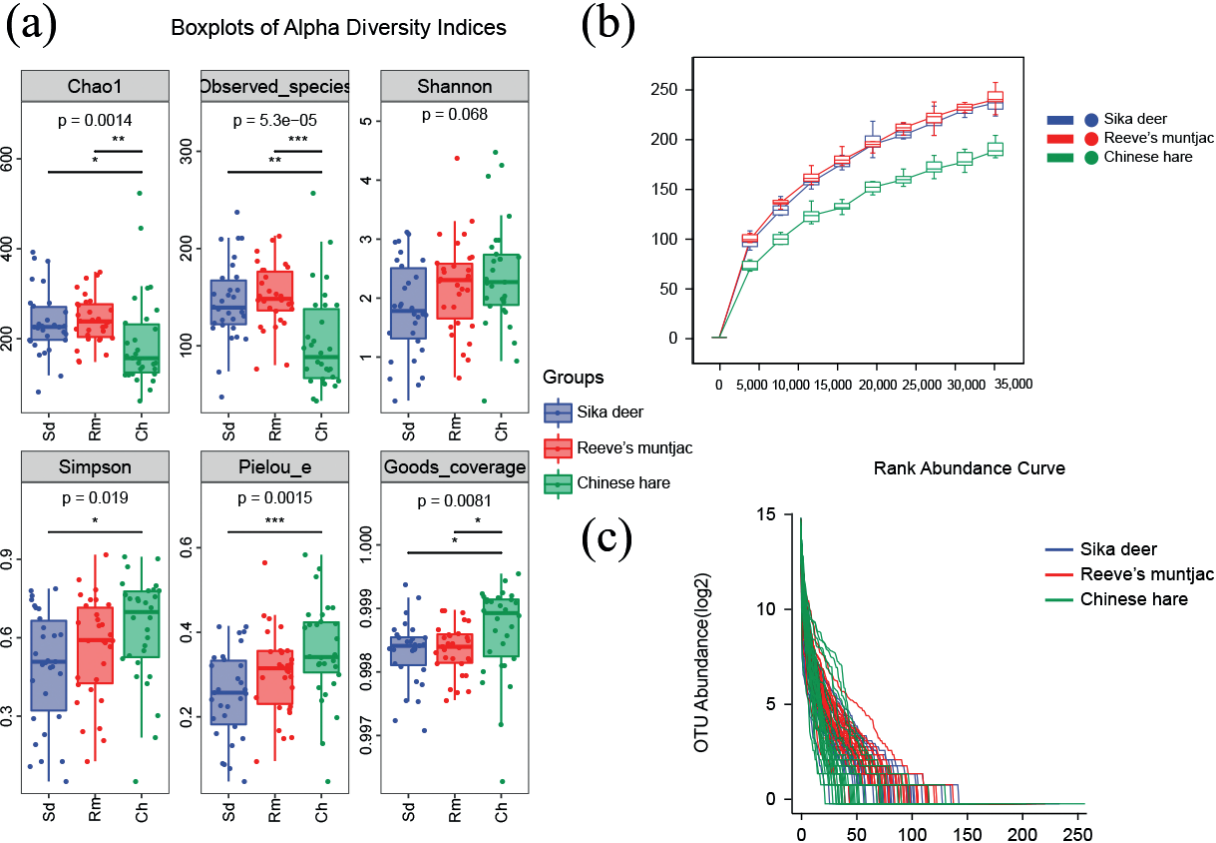
**a** box-plot of the alpha diversity index. In each panel, the abscissa is the group, and the ordinate is the value of the corresponding alpha diversity index **b** sample rare faction curves **c** rank abundance curve. The abscissa is the sequence number of OTUs arranged according to the Abundance size. The ordinate is the abundance value of each OTU in this grouping by Log2 log transformation (Ch: Chinese hare; Sd: Sika deer; Rm: Reeve’s muntjac).

Alpha diversity reflects the abundance and diversity of species communities. The Chao1 and Observed species indices showed the highest community richness was Reeve’s muntjac (Chao1 index; Reeve’s muntjac = 242.46, Sika deer = 236.52, Chinese hare = 192.03, on average). The Shannon and Simpson indices showed the highest community diversity was Chinese hare (Shannon index; Chinese hare = 2.36, Reeve’s muntjac = 2.21, Sika deer = 1.82, on average), with no significant differences (*P* > 0.05). The goods coverage of 0.998 indicated that an average of 99% of the species were annotated (Fig. 4a; Table 2). Rarefaction curves describe the increase in species diversity as the sample size increases. It is crucial to point out that the characterization of species diversity was considered very reliable since the depth of rarefaction applied (35,000) was found to be sufficiently satisfactory (e.g., rarefaction curves had already reached a plateau at ~35,000 sequences in all samples) (Fig. 4b). The rank abundance curve reflects the richness and evenness of species in the sample through the flatness. The evenness of community composition of sika deer and Reeve’s muntjac was higher, while the lowest of Chinese hare (Fig. 4c).

**Table 2.** Alpha diversity index among three sympatric species including sika deer, Reeve’s muntjac, and Chinese hare.

Sample ID	Sika deer						Reeve’s muntjac						Chinese hare					
	Chao1	Goods _coverage	Observed _species	Pielou_e	Shannon	Simpson	Chao1	Goods _coverage	Observed _species	Pielou_e	Shannon	Simpson	Chao1	Goods _coverage	Observed _species	Pielou_e	Shannon	Simpson
001	280.705	0.998038	170.2	0.40159	2.97535	0.760442	221.216	0.998458	147.5	0.169581	1.22164	0.254821	234.755	0.998217	141.9	0.384791	2.75059	0.766172
002	229.404	0.998361	154.1	0.257889	1.87412	0.511389	223.198	0.998492	144.1	0.22161	1.58889	0.400989	144.924	0.998847	93.3	0.348889	2.28297	0.717446
003	226.705	0.998481	131.2	0.265175	1.86516	0.504481	148.447	0.998978	76.4	0.152733	0.955071	0.242638	122.406	0.999139	66.2	0.442545	2.67587	0.781497
004	83.0718	0.999378	47.5	0.04717	0.262596	0.050021	199.96	0.998608	139	0.4334	3.08469	0.822047	190.257	0.998583	97.2	0.342704	2.26105	0.678976
005	260.619	0.998344	140.5	0.41492	2.95988	0.745168	343.206	0.997691	213.2	0.565935	4.37752	0.917577	132.754	0.999174	64.7	0.442643	2.66008	0.786546
006	197.435	0.998546	118.2	0.265982	1.83103	0.542388	265.68	0.998131	178.1	0.247444	1.84993	0.420895	244.217	0.998111	141.9	0.27918	1.99433	0.632656
007	231.011	0.998546	109.6	0.321788	2.17979	0.606797	198.505	0.998824	126.7	0.148754	1.03889	0.208596	109.736	0.999253	59.4	0.34015	2.00346	0.532667
008	186.477	0.998679	128.3	0.132388	0.926968	0.19294	173.466	0.998816	119.8	0.095464	0.658982	0.127527	223.278	0.99833	109.7	0.32719	2.21691	0.679038
009	176.872	0.998759	121.7	0.227483	1.57562	0.515318	201.798	0.998665	123.3	0.357705	2.48433	0.745497	147.016	0.999049	62.2	0.32465	1.9334	0.602632
010	226.03	0.998381	138.1	0.243521	1.73098	0.492527	284.977	0.998219	170.6	0.336498	2.49463	0.590127	145.945	0.998992	83.5	0.320825	2.04777	0.5727
011	224.427	0.998378	150.5	0.177627	1.28473	0.291992	230.017	0.998401	151.9	0.307556	2.22853	0.589744	199.096	0.998449	125.3	0.333101	2.32102	0.750859
012	280.773	0.997847	183.2	0.340063	2.55598	0.716689	244.458	0.998393	151.8	0.297168	2.15298	0.502685	290.705	0.998009	142.6	0.418089	2.99021	0.757656
013	220.095	0.998438	135.6	0.242773	1.71921	0.485882	239.338	0.99831	135.8	0.292677	2.0731	0.64653	264.841	0.9981	152.8	0.411819	2.9874	0.809688
014	328.541	0.997802	209.9	0.343807	2.65167	0.673406	217.883	0.998549	146.6	0.32392	2.33042	0.568292	138.488	0.999117	76.9	0.42012	2.63072	0.749443
015	381.036	0.997089	237.8	0.336105	2.65258	0.723663	150.282	0.998963	80.2	0.218794	1.38347	0.43972	132.877	0.999191	76.6	0.198498	1.2422	0.305309
016	334.337	0.997563	191.8	0.413	3.13158	0.781254	277.335	0.997947	181.3	0.440708	3.30546	0.785065	120.473	0.999199	68.5	0.32578	1.9862	0.504604
017	207.833	0.998586	107.7	0.282636	1.90708	0.611331	348.937	0.997558	209	0.401727	3.09589	0.765458	87.2741	0.999438	43.5	0.3264	1.77444	0.551934
018	393.986	0.997237	210.8	0.292397	2.257	0.609557	274.614	0.998214	178.1	0.318902	2.38396	0.58144	445.677	0.997171	207.1	0.583434	4.48816	0.909631
019	233.482	0.998353	158.1	0.323043	2.35938	0.639344	260.417	0.998234	165	0.310687	2.28763	0.651905	167.976	0.998648	98.6	0.239998	1.58907	0.424112
020	170.192	0.998677	118.9	0.07857	0.541529	0.109537	210.012	0.9986	148.8	0.21039	1.51822	0.361145	155.937	0.998963	75.2	0.299848	1.86801	0.523298
021	198.276	0.998529	127.2	0.162252	1.134	0.299678	237.626	0.998549	147.7	0.32251	2.32356	0.61479	107.632	0.999233	60.8	0.419374	2.48264	0.744613
022	373.107	0.997535	211.1	0.400956	3.09591	0.787719	281.474	0.998185	143.1	0.355952	2.54866	0.668624	121.692	0.999142	64.6	0.25249	1.51731	0.452847
023	242.856	0.998137	145.9	0.195825	1.40746	0.387288	253.578	0.998359	153.8	0.294375	2.13846	0.59175	524.096	0.996277	257.2	0.531387	4.2544	0.873609
024	194.829	0.998489	123.5	0.202812	1.40898	0.463457	226.685	0.998458	137.9	0.414342	2.94376	0.747215	162.056	0.99889	105.8	0.138661	0.932367	0.218141
025	228.774	0.998424	157.8	0.381325	2.78406	0.712446	316.573	0.997734	187.1	0.35747	2.69716	0.728621	157.534	0.999094	76.6	0.459812	2.8768	0.781035
026	165.272	0.998938	108.6	0.256367	1.73316	0.499269	200.122	0.998572	119.8	0.269879	1.86281	0.449013	62.196	0.999548	45.3	0.047218	0.259645	0.051564
027	118.54	0.999171	73.7	0.150522	0.933483	0.232503	165.642	0.998935	115.2	0.224942	1.54008	0.352465	316.768	0.997776	171.4	0.550031	4.08039	0.905171
028	216.523	0.998398	133.9	0.089286	0.630779	0.129084	241.525	0.998131	156.7	0.35582	2.59425	0.723594	176.083	0.998725	101.4	0.270131	1.80004	0.452913
029	274.14	0.998097	171.2	0.226096	1.67727	0.435829	300.379	0.997947	184.8	0.326165	2.45546	0.660694	313.133	0.997844	172.6	0.458045	3.40363	0.799484
030	210.264	0.998441	152.1	0.089529	0.64893	0.128594	336.477	0.997683	197.7	0.353038	2.69256	0.689019	121.149	0.999105	81.2	0.424873	2.69476	0.734457
Average	236.520	0.998321	145.6	0.252097	1.82321	0.488000	242.461	0.998353	151.0	0.304205	2.21037	0.561616	192.032	0.998654	104.1	0.355422	2.36686	0.635023

**Figure 5.**
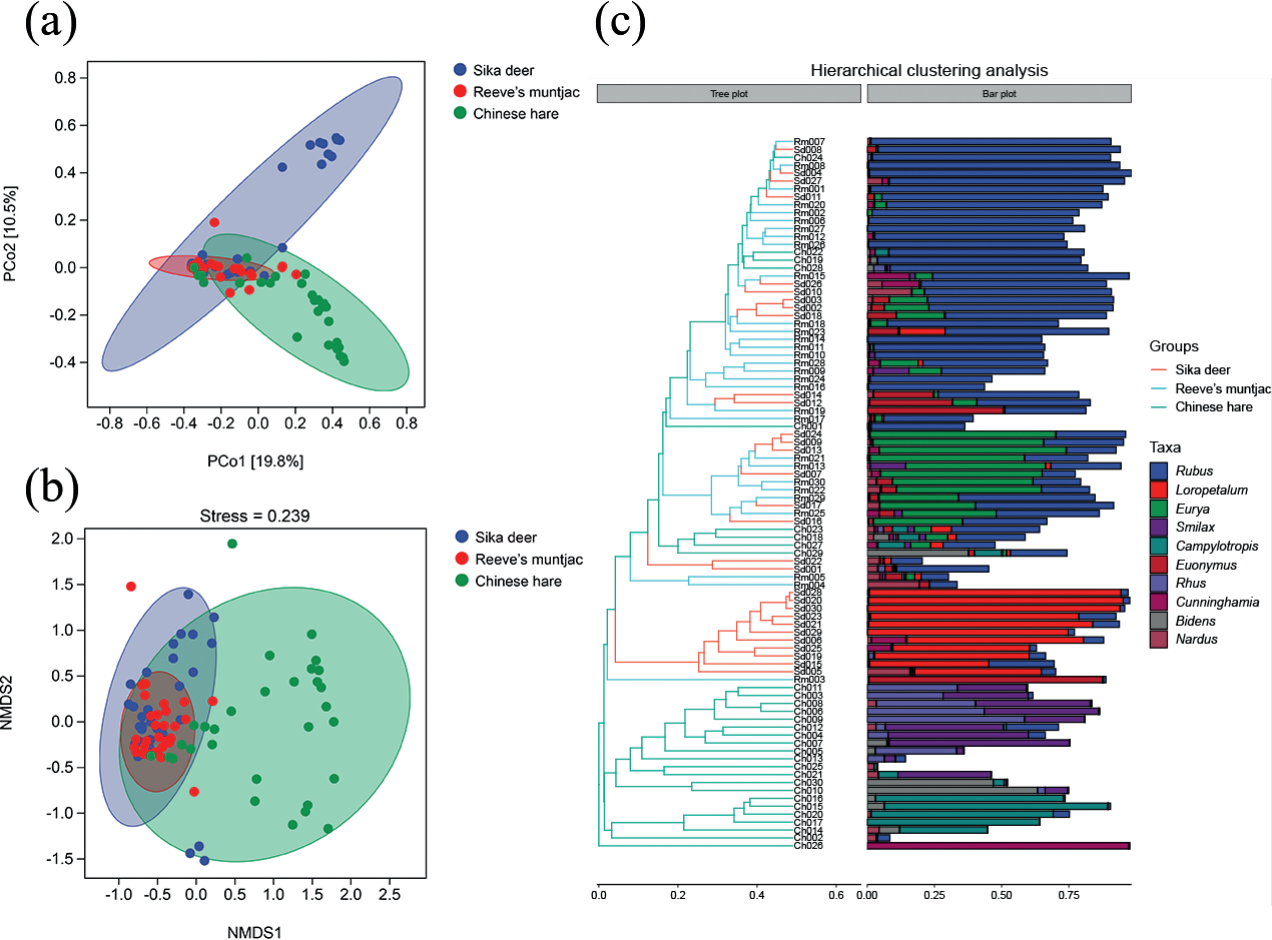
**a**PCoA analysis chart, in which each point represents a sample **b**NMDS analysis chart. Diagram analysis with 95% confidence ellipse **c** hierarchical clustering diagram. Analysis of the hierarchical clustering tree diagram and the stacked bar diagram of the top 10 genera in abundance (Ch: Chinese hare; Sd: Sika deer; Rm: Reeve’s muntjac).

We assessed the beta diversity using the Bray–Curtis distance. When the distance between the samples was smaller, the species-composition structure was more similar, and the PCoA diagram and NMDS analysis revealed the similarity of the composition of the diet between sika deer and Reeve’s muntjac (Fig. 5a, b), which was consistent with our expected results. In addition, a hierarchical clustering heat map was convenient for the intuitive identification of the species present in corresponding samples; *Rubus* had the highest abundance among the three species. The tree plot indicated that there was much similar distance in the samples between sika deer and Reeve’s muntjac but less similar with Chinese hare (Fig. 5c).

**Figure 6.**
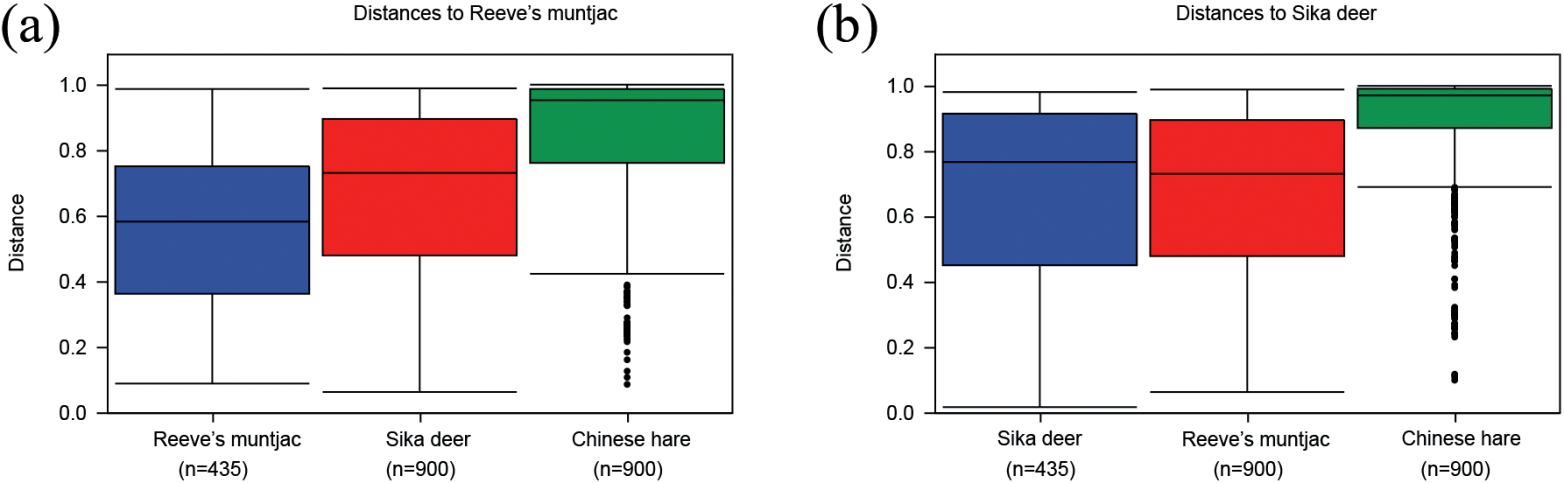
Intergroup difference analysis **a** shows the boxplots of the distances between samples in the sika deer group and the distances between samples in this group and samples in other groups **b** shows the boxplots of the distances between samples in Reeve’s muntjac group and the distances between samples in this group and samples in other groups.

The intergroup difference analysis shows the difference between the intragroup and intergroup sample distances. Compared with Reeve’s muntjac, the intragroup distance of Reeve’s muntjac was smaller than the intergroup distance of sika deer and Chinese hare (Fig. 6a). While compared with sika deer, the intragroup distance of sika deer was slightly higher than the intergroup distance of Reeve’s muntjac (Fig. 6b). With different species composition, the difference between the intragroup should be smaller than the intergroup. In comparison with the NMDS, this phenomenon was speculated to be the fact that the diet composition of Reeve’s muntjac was much similar to those of sika deer, and the Reeve’s muntjac has more forage plant diversity.

By analyzing the niches of the three sympatric herbivorous animals, we found that the highest niche breadth was Chinese hare (~7.78), followed by sika deer (~4.53) and Reeve’s muntjac (~3.44). The niche overlap index between sika deer and Reeve’s muntjac was 0.83, sika deer and Chinese hare was 0.62, and Reeve’s muntjac and Chinese hare was 0.69. The overlap index ranges from 0 to 1, where 0 indicates that the food ranges do not overlap at all, and 1 indicates that the food ranges overlap entirely. Our results suggested that Reeve’s muntjac and sika deer have the highest diet overlap (Table 3).

**Table 3.** The dietary niche overlap and Observed niche overlap index among the three sympatric species.

	Dietary niche breadth	Interspecific comparation	Observed niche overlap index
Sika deer	4.53	Sika deer vs Reeve’s muntjac	0.83
Reeve’s muntjac	3.44	Sika deer vs Chinese hare	0.62
Chinese hare	7.78	Reeve’s muntjac vs Chinese hare	0.69
–	–	Sika deer vs Reeve’s muntjac vs Chinese hare	0.68

## ﻿Discussion

### ﻿The food composition of the three herbivorous animals

Quantitative analysis is of great significance for the families, genera, and species of the herbivores’ diet. Liu (2012) investigated the diet of wild northeast sika deer in winter and found the sik deer feed on 35 plant species belonging to 25 genera in 17 families. Liu (2020) showed that 131 plants, including nine herbs, 31 shrubs, and eight trees, were foraged by South China sika deer in the Tianmu Mountains. One hundred and thirty-nine food items were identified in the feces of sika deer in our study. Comparative analysis shows that the metabarcoding method based on high-throughput sequencing provides more detailed forage plant information.

Yao et al. (2010) found that the sika deer in TNNR mainly eat *Homonoiariparia*, *Fallopiamultiflora*, *Lespedezabicolor*, *Puerariamontana*, and *Viciafaba*, and other species, and then especially fed on young leaves and shoots (Li et al. 2014). Compared with our study, the dominant forages in the feces of sika deer were *Rubuschingii*, *Loropetalumchinense*, *Euryajaponica*, *Camelliajaponica*, *Euonymusgrandiflorus*, and *Phyllostachysedulis* in winter; these are all Chinese medicinal herbs, which ensure the prevention of various diseases throughout the life cycle of sika deer (Yao et al. 2010; Ye 2015). The differences may be attributed to geographical and seasonal differences. The vegetation condition varied according to the different regions, which means that diverse forage plants are available for the sika deer.

In seasons when plant resources are scarce, sika deer will choose to eat non-favorable plants or the available food resources at the moment. Studies on Japanese sika deer showed that they mainly choose their favorite deciduous species from summer to autumn, such as *Cornuscontroversa*, and *Quercus* sp., but from early winter to spring, non-favored herbaceous and tree species, such as *Juncusdecipiens* and *Cryptomeriajaponica*, will be foraged (Nakahama et al. 2020). Therefore, sika deer have a diverse diet and feed on various plants in different seasons (Yao et al. 2010; Wang et al. 2019). In the TNNR, oaks included *Quercusacutissima*, *Q.aliena*, *Q.chenii*, *Q.fabri*, and *Q.serrata* (Jiang 2009). Significantly, oak leaves, which are rich in tannins and toxic to most mammals, including cattle (Doce et al. 2009), are conversely found to increase the reproductive rate and fawn survival rate of sika deer in captive breeding by some farmers (Xing et al. 2022). Oak leaves are essential for maintaining healthy sika deer in wild and farmed populations. However, we found a lower abundance in *Quercus* for three herbivorous mammals’ diets. We speculated this might be related to deciduous *Quercus* in winter or the gestation period of female sika deer, even though some studies claimed that tannins were not toxic to sika deer because of their rumen microbes and fermentation processes (Li et al. 2013). There are differences in the forage plants of South China sika deer in different seasons and regions.

In our study, we identified most of the forage species; however, someof the forage species that we identified were not previously known from Jiangxi Province. It may be difficult to identify all forage plants using a single gene fragment, and continuous succession of plant communities caused by invasive species adds a level complication to this. Therefore, it is necessary to add auxiliary barcodes as well as strengthen the overall investigation of potential food resources in the reserve. The construction of a database of plant species barcodes for the Taohongling Sika Deer Reserve would provide a reference and source of sequence alignments. Such as database would allow for a more accurate determination of the diets of herbivores and allow for better comparisons of the diets of sympatric herbivores.

### ﻿Interspecific niches

Competition theory indicates that the greater the overlap of resources between species, the greater the competition coefficient because of the widespread use of niche overlap to estimate competition for resources (Colwell and Futuyma 1971). Tibetan red deer have a similar diet to sympatric ungulates, which inevitably leads to interspecific conflicts in food use (Lu et al. 2020). However, spatial-temporal variations in dietary consumption of the two dominant rodent species on Mount Kilimanjaro, Tanzania, have been found serve as a mechanism of resource portioning that enable these species to coexist with a niche overlap (Mulungu et al. 2011; Thomas et al. 2022); this contradicts the key assumption of competition theory. Thus, we cannot limit the research on interspecific competition in sympatric species to trophic ecological niches, and spatial-temporal dimensions should also be considered. For sika deer in the TNNR, we do not know the exact reasons for spatial variations in dietary overlaps with its two sympatric herbivores. We suggest that a cautious approach is required to interpret the high dietary overlaps and their implications for competitive interactions among the three studied herbivores in the TNNR. As pointed out by others (Belovsky 1986; Jenkins and Wright 1988; Gordon and Illius 1989), high dietary overlaps may not necessarily imply competition (Andersen et al. 2017) and may simply indicate that the food item is sufficient, permitting sympatric species to share resources. In the TNNR there are increasing populations of sympatric wild boar and Reeve’s muntjac. Sika deer face competition stress in space and food resources, especially when food is scarce in winter (Li et al. 2014). Thus, even when the determinants of competition mechanisms are uncertain, competition does exist and its important role among species cannot be denied.

Competition among sympatric species is mostly expressed as a compensatory mechanism in ecological niches when species are similar in one dimension, they differ on another. Food resources, habitat, and temporal partitioning are the most common dimension partitioned (Bagchi et al. 2003). For example, high dietary overlap among the species may result in niche differentiation (Reitz and Trumble 2002; Yin et al. 2007; Cao et al. 2009); large herbivores are forced to expand their food range to avoid competition during periods of food scarcity (Noor et al. 2013). In northeastern China, red deer tend to increase their browsing intensity to maintain their high food intake, but sika deer meet their relatively constant food intake and potential nutritional requirements by increasing their bite diameter in winter. This reflects the short-term foraging strategies by sharing similar foods with the sympatric ungulates (Zhong et al. 2020). Currently, we do not know the competition mechanism of sika deer and further studies are needed to determine the coexistence mechanism with its sympatric species.

In our study, we found the niche breadth of the sika deer was higher than the Reeve’s muntjac. Optimal forage theory suggests that preference and palatability will be selected for the animals in abundant food periods. While in a period of scarce food resources, feeding generalization will occur by selecting different forage plants (Belovsky 1978). It remains to be studied whether the higher dietary niche breadth of sika deer results from the physiological characteristics of digesting a wide range of foods or to avoid competition. Moreover, the niche breadth of Chinese hare was larger than sika deer and Reeve’s muntjac. We speculate that Chinese hare, as opportunistic feeders, have a broader range of forage plants but consume less due to their smaller body size. The diet composition of Chinese hares includes trees, shrubs, and herbs, but this may be due to a passive and random proximity foraging strategy or even the indirect ingestion from the process of grinding teeth.

## ﻿Conclusions

The South China sika deer is the most endangered among the three remaining subspecies of sika deer in China. In our study, sika deer and Reeve’s muntjac showed a higher overlapping index of niche. Reeve’s muntjac may affect the survival of the sika deer due to the shortage of food resources in winter. We speculated that potential competition probably occurs in two cervid species. In addition, the growth of the secondary vegetation has accelerated in the reserve, and the decline of suitable habitats is a serious threat to the growth of the sika deer population. It is urgent to strengthen habitat management, improve habitat quality, and study forage plants. It is also necessary to provide food for sika deer and other wildlife through artificial planting during food shortages and dry seasons. Further studies need to establish local DNA databases to identify the forage plants and introduce the auxiliary barcoding to solve accurate species-level diet composition. Overall, our study determined the diet composition and interspecific niches of South China sika deer and its sympatric Reeve’s muntjac and Chinese hare. These result should be helpful to facilitate habitat improvements and artificial planting, monitor forage resources, and conserve biodiversity, and manage the reserve.

## Supplementary materials

Supplementary material 1 Dietary of Sika deer, Reeves՚ muntjac and Chinese hare Data type data (excel document) https://binary.pensoft.net/file/814242This dataset is made available under the Open Database License (http://opendatacommons.org/licenses/odbl/1.0/). The Open Database License (ODbL) is a license agreement intended to allow users to freely share, modify, and use this Dataset while maintaining this same freedom for others, provided that the original source and author(s) are credited.Dandan Wang, Xiaolong Hu, Minling Li, Jie Liu, Ming Tang, Wuhua Liu, Jianwen Zhan, Yongtao Xu,Weiwei Zhang
